# 3D-Printed Models versus CT Scan and X-Rays Imaging in the Diagnostic Evaluation of Proximal Humerus Fractures: A Triple-Blind Interobserver Reliability Comparison Study

**DOI:** 10.1155/2022/5863813

**Published:** 2022-06-13

**Authors:** Gianluca Puglisi, Marco Montemagno, Regina Denaro, Giuseppe Condorelli, Vincenzo Fabrizio Caruso, Andrea Vescio, Gianluca Testa, Vito Pavone

**Affiliations:** Department of General Surgery and Medical Surgical Specialties, Section of Orthopaedics and Traumatology, University Hospital Policlinico-San Marco, University of Catania, Catania, Italy

## Abstract

**Background:**

Proximal humerus fractures (PHFs) are one of the most frequent fractures in the elderly and are the third most fractures after those of the hip and wrist. PHFs are assessed clinically through conventionally standard imaging (X-ray and computed tomography (CT) scans). The present study aims to conduct the diagnostic evaluation and therapeutic efficacy of the 3D-printed models (3DPMs) for the PHFs, compared with the standard imaging.

**Objectives:**

In terms of fracture classification and surgical indication, PHFs have poor interobserver agreement between orthopedic surgeons using traditional imaging such as X-rays and CT scan. Our objective is to compare interobserver reliability in diagnostic evaluation of PHFs using 3DPMs compared to traditional imaging.

**Methods:**

The inclusion criteria were elders aged >65 years, fracture classification AO/OTA 11 B and 11 C, and no pathological fractures or polytrauma. In addition, 9 PHFs were assessed by 6 evaluators through a questionnaire and double-blinded administered for each imaging (X-ray and CT scan) and 3DPMs for each fracture. The questionnaire for each method regarded Neer classification, Hertel classification, treatment indication (IT), and surgical technique (ST). Interobserver reliability was calculated through the intraclass correlation coefficient (ICC).

**Results:**

Nine patients with PHF were included in the study (66% female). The Neer and Hertel classifications between imaging types had similar ICC values between raters with no statistical differences. IT reliability using CT scan and 3DPMs (ICC = 1; (*p*=0.116)) assessed better agreement compared with X-rays IT. The ST reliability using 3DPMs (ICC = 0.755; *p*=0.002) was statistically superior to traditional imaging (ST-RX ICC = -0.004 (*p*=0.454); ST-CT ICC = 0.429 (*p*=0.116)).

**Conclusion:**

Classification systems like Neer and Hertel offer poor reliability between operators. The 3DPMs for evaluating diagnostics are comparable to CT images but superior to the surgical technique agreement. The application of 3DPMs is effective for preoperative fracture planning and the modeling of patient-specific hardware.

## 1. Introduction

Proximal humeral fractures (PHFs) are one of the most frequent fractures among the elderly and are the third most fractures after hip and wrist fractures [1, 2]. PHFs account for 45% of upper limb fractures and 4-5% of all the overall fractures. In addition to the typology of the fracture, decision-making is also influenced by underlying patient and surgeon-related factors. However, no entirely satisfactory fracture classification is available to serve as a guide to modern treatment and predict an outcome. While the Neer classification [3] has been the most widely used, it has poor inter and intraobserver reliabilities [4]. The anatomic system is based on the degree of involvement and displacement of the four major fracture segments on plain radiographs; but it does not include some of the more recently described fracture types, which may have a more favorable prognosis: impacted-valgus fractures [5, 6], varus fractures [7], the various subtypes of anterior, and posterior fracture-dislocations [8]. Although refinements [9] and more detailed systems [10, 11] have been applied, none has gained the level of acceptance of the Neer classification [2, 4].

Radiographs should be used as the first-line study to evaluate PHF [12]. Neer's original study suggested that evaluation should be based on an anteroposterior radiograph and a scapular *Y* view [3]. However, subsequent work suggests an additional axillary view [3], as it is useful for greater and lesser tuberosity fractures, head-split fractures, and glenohumeral dislocations. Computed tomography (CT) is effective in evaluating proximal humeral fractures, especially in complex cases. CT helps to improve preoperative planning, especially when internal fixation is used during arthroplasty. CT scan is used to detect occult fractures, posterolateral humeral head fractures, lesser tuberosity fractures, and head-split fractures [13]. Although radiologists are familiar with basic fracture around the shoulder, in this area, certain fracture types are particularly relevant, clinically controversial, and require classification management [14].

Surgical treatment of PHFs has several options such as open reduction and internal fixation (ORIF) with plate in 2-3 fragments reducible fractures; intramedullary nailing is a great option for the fractures of humeral neck; external fixator or K-wires are useful for complicated cases, when open reduction have too many risks; shoulder arthroplasty is chosen for displaced 3-4 fragments fractures or PHF at risk of avascular necrosis in the elderly [15–17].

Managing PHFs are not free from risks. In conservative treatment, some possible complications are represented by delayed union or nonunion and osteonecrosis of the humeral head.

Surgical treatment complications described in literature are iatrogenic rotator cuff lesions, secondary displacement, partial or total osteonecrosis, screw penetration of the articular surface, nonunion, infection, cut out and aseptic loosening of implant devices, axillary nerve injury, iatrogenic fracture, heterotopic ossification, and dislocation [18, 19].

It is also reported by Biz et al. a late onset axillary artery and deep vein thrombosis in PHFs dislocation [20].

With the rapid development of technology, orthopedic has applied digital technology for clinical services [21]. With the emergence of 3D printing technology (3DPT), the digital preoperative design and simulation can be transferred from the virtual stage to the reality stage. Technology helps surgeons to perform correct diagnoses and conduct individual operation plans, which significantly improve the safety and the effectiveness of surgery. With the aid of 3DPT, patient-specific implant designs are vital for epoch-making shift [22].

In recent years, 3DPT has gradually been applied in the medical field owing to its ability to display fractures [23]. In addition, the 3D imaging on PHFs significantly increases the number of surgical decisions and the number of ORIF indications when compared to radiographs alone or in combination with CT imaging [24]. Other studies claim that 3DPT has not improved classification reliability when compared to classic imaging investigations [25, 26], since a complex classification is not easily translated from images.

The present study aims to evaluate the diagnostic and therapeutic efficacies of 3DPT compared to the standard imaging (X-rays and CT scan) in PHFs, by measuring the interobserver agreement differences among orthopedic surgeons in the most used classifications and the treatment indications.

## 2. Materials and Methods

### 2.1. Demographic Data

This was a triple-blind trial study that included 9 cases with PHF, in which patients were recruited from January 2015 to January 2021. The inclusion criteria were age >65 years, fracture classification AO/OTA 11 B, and 11 C. The exclusion criteria were age <65 years, pathological fracture, metabolic bone disease, and polytrauma.

**Figure 1:**
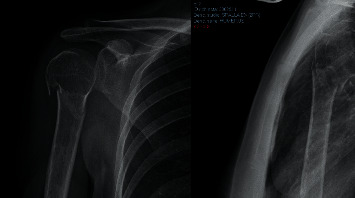
A standard anterior-posterior and axillary X-ray view of a PHF.

A diagnostic and therapeutic evaluation of PHFs was proposed using X-ray images (Figure 1), CT scans, and 3D-printed models (3DPMs) separately. All PHFs were independently examined and assessed by six different orthopedic surgeons working in our orthopedic department. All evaluators had at least five years of shoulder surgical experience and were competent in the scoring systems.

PHFs were assessed by raters through a questionnaire for each imaging and 3DPM for each fracture. The test was administered randomly by a second operator blinded to the questions. A third blinded operator processed collected data statistically.

Patients had given voluntary informed consent to participate in this study research.

### 2.2. Evaluation of Diagnostic Tests

**Figure 2:**
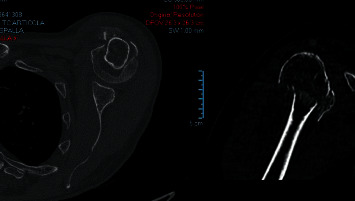
CT scan layer of a PHF in axial and frontal planes.

**Figure 3:**
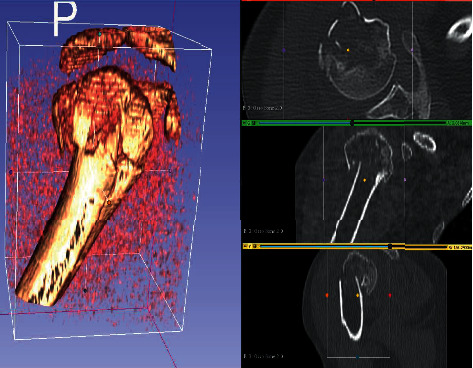
Slicer software user interface showing the segmentation process of a 3D digital model from a CT scan of PHF.

**Figure 4:**
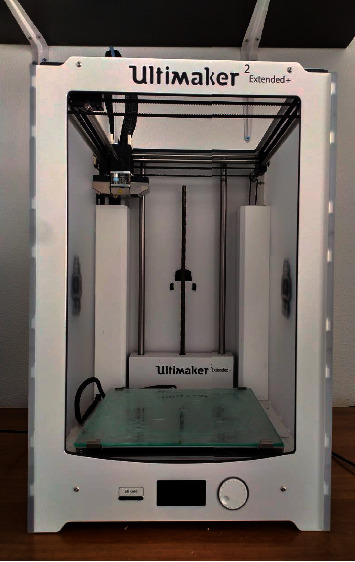
Ultimaker 2+ extended 3D printer.

**Figure 5:**
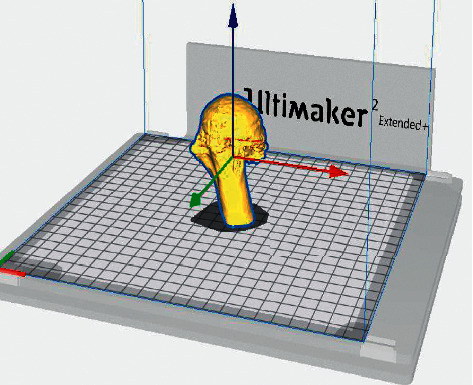
Final planning of the 3D model of PHF ready for printing.

**Figure 6:**
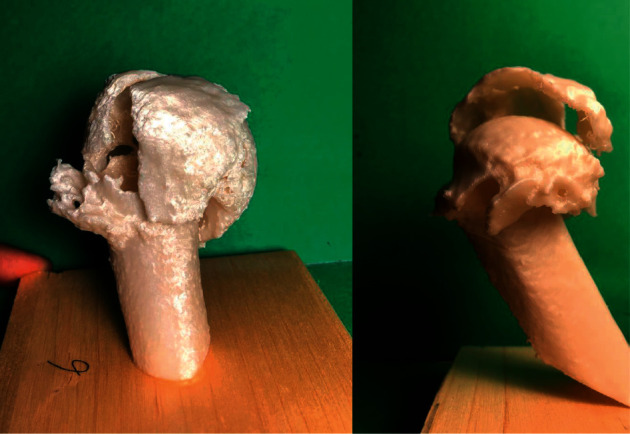
3DPMs of PHF used in the following study.

Each PHF was rated with the following medical information:

1.  Anteroposterior and “scapular Y” X-ray views (Figure 1)
2.  CT scans (Optima CT660) (Figure 2) were acquired with 1 mm layer thickness, 1 mm layer interval, and a voltage of 120 kV without using volume rendering
3.  CT DICOM files were subsequently processed using a medical 3D processing software (Slicer version 4.11.20210226) (Figure 3) to construct 3DPMs. This work included understanding of the intact structures of the proximal humeral and bones around the shoulder joint, segmentation by applying the region growing function to separate the bones and soft tissues, and distinguishing the PHF from the normal scapula and clavicle; segmentation to define the fracture fragments of humeral and build a proximal humeral 3D digital model. The outcome of the test was imported into rapid prototyping equipment (Ultimaker 2+ extended, https://www.ultimaker.com) (Figures 4 and 5) for the 3D printing of a final solid fracture model (Figure 6).

Each fracture was presented in the same way: gender, years, and fractured side. A blind operator presented data randomly to evaluators, which classified imaging and 3DPMs according to

-  Neer classification
-  Hertel classification
-  Treatment indication, and
-  Surgical technique

### 2.3. Imaging Severity Assessments

Neer classification: the Neer classification evaluates the fracture of the four proximal humeral fragments: greater tuberosity, lesser tuberosity, surgical neck, and anatomical neck. The classification helps to understand the numbers of the fragments, the diastasis, and the angle of bone fragments. Neer classification considered fracture to be unstable if angulation exceeded 45 degrees or if the fracture was displaced by more than 1 cm [3].

Hertel classification: “Lego classification” assesses the risk of head ischemia based on model fracture, medial hinge integrity, and dorsomedial metaphysical extension length [11]. A total of 12 basic fracture patterns of the proximal humeral was identified: 6 fractures dividing the humerus into two fragments, 5 fractures dividing the humerus into three fragments, and a single fracture pattern dividing the humerus into four fragments. According to Hertel, anatomical neck fractures increased the risk of avascular necrosis.

### 2.4. Treatment Indication

Each fracture was assessed by operative treatment or nonoperative treatment. The indications for nonoperative treatment for the patients included in the study were nondisplaced fractures, valgus impacted fractures, and mildly displaced fractures (<1 cm, <45°) [19].

### 2.5. Surgical Technique

Evaluators could choose different surgical treatment for each PHFs: ORIF using plate and screws, intramedullary nailing, pinning, arthroplasty with reverse shoulder replacement, anatomic shoulder replacement, and external fixator. Patients included in the study were treated with ORIF with plate (77%), intramedullary nailing (11%), and arthroplasty (11%).

### 2.6. Statistical Analysis

Data were collected with a questionnaire using Microsoft Excel (version 16.0.14527.20162). Continuous data are presented as means and standard deviations. The intraclass correlation coefficient (ICC) (two-way random effects model and single-measure reliability) was performed to evaluate the interobserver agreement. According to the Koo and Li [27] guideline, agreement below 0.50 was considered “poor,” agreement between 0.50 and 0.74 was “moderate,” agreement between 0.75 and 0.89 was “good,” and above 0.90 was considered “excellent” (Table 1). The standard Student' *t*-test was used to compare ICC scores, with a significance level of *p* < 0.05 and 95% confidence interval (CI). All statistical analyses were performed using the SPSS software (IBM®).

## 3. Results

Nine patients with PHF were considered eligible and included in the study: 6 females (66%) and 3 males (33%). The mean age was 68 years (range: 54–74 years). Data were collected by questionnaire, and the following results were presented.

### 3.1. Neer Classification Interobserver Reliability

In general, the Neer classification (NC) scores among operators had “good” reliability values according to ICC in each diagnostic method. NC with X-rays imaging between observers was significant, ICC = 0.740 (*F*_8,40_ = 3.843; *p*=0.002). The same result was found for NC with CT scan, ICC = 0.792 (*F*_8,40_ = 4.817; *p*=0.001), and NC with 3DPMs, ICC = 0.770 (*F*_8,40_ = 4.347; *p*=0.001) (Table 2).

### 3.2. Hertel Classification Interobserver Reliability

Same results were obtained for the Hertel classification (HC) reliability, with higher ICC values compared to NC and “excellent” ICC value for 3DPMs. HC with X-ray had significant scores: ICC = 0.852 (*F*_8,40_ = 6.735; *p*=0.001). Similar result was obtained by HC with CT scan: ICC = 0.806 (*F*_8,40_ = 5.146; *p*=0.001) and HC with 3DPMs : ICC = 0.922 (*F*_8,40_ = 12.845; *p*=0.001) (Table 2).

### 3.3. Treatment Indication Interobserver Reliability

The treatment indication (IT) with X-ray evaluation between operators had lower agreement, ICC = 0.467 (*F*_8,40_ = 1.875; *p*=0.091). Different results were obtained by CT scan IT: ICC = 1 (*F*_8,40_ = 1.000; *p*=0.451) and 3DPMs IT: ICC = 1 (*F*_8,40_ = 1.000; *p*=0.451) (Table 3).

### 3.4. Surgery Technique Interobserver Reliability

Surgery technique (ST) reliability resulted higher using 3DPMs with statistical significance resulting ICC = 0.755 (*F*_8,32_ = 4.089; *p*=0.002). No significance and lower values were observed in the interobserver reliability in ST with X-rays: ICC = −0.004 (*F*_8,40_ = 0.996; *p*=0.454) and ST with CT scan: ICC = 0.429 (*F*_8,40_ = 1.751; *p*=0.116) (Table 3).

## 4. Discussion

This study is one of the first to use 3DPT to study PHFs, with an avant-garde method for diagnostic and surgical treatment. Treatment of PHFs can be conservative or surgical. However, surgery is the most recommended for fractures with marked displacement or young people with Neer 3-4 part fracture [28–30].

The most used treatment in literature is the conservative, especially in elderly with poor baseline function. The surgery treatment must be preferred in young patients and complex fractures for an early functional recovery. Internal fixation and arthroplasty are the most employed strategies to manage complex fractures. There is a controversial treatment for 3 fragments of PHFs, neck fractures in elderly, and the surgery treatment with a plate for three fragments of PHFs [15, 31]. This study found a good interobserver reliability among imaging modalities in the PHFs classification, and it was statistically significant. Among various methods, a good correlation was found between all the operators for the Neer and Hertel classifications. CT scan and 3DPMs resulted in general with a higher interobserver agreement compared to X-ray evaluation of PHFs. The most relevant result revealed that the interobserver correlation of 3DPMs was better than CT for the surgery technique proposed.

Presently, 3DPT has been demonstrated as a useful tool in the orthopedic field. The surgery team and other clinical professionals can use 3DPT, a real fracture printed model, to improve the performance of the treatment. 3DPMs can also be presented to the patients and their family members to facilitate their communication with surgeons. Traditional imaging tests fail to present the advantages of the configuration of the fracture, the deformities, and the sense of depth in 3DPMs [29, 30].

Moreover, studies of Wu et al. [30] demonstrated that 3DPMs help surgeons to better select the correct and appropriate size and length of the plate and screws, performing accurate surgical plans for treating complicated PHFs. Medical teaching should apply 3DPT to increase the cultural baggage/preparation of the resident surgeons. Importantly, the total number of fractures in three and four parts significantly increased when 3D imaging was introduced. Thus, 3DMPs imaging increased the perception of the complexity of the fracture model [30].

Majed et al. [32] suggested that current PHFs classifications have poor intra and interobserver reliabilities, making the decision of treatment difficult to perform. In our study, the interobserver reliability for the evaluation of 3DPMs stands out among other imaging methods, especially for the treatment with surgery. The choice of the classification and the type of treatment is consistent with previous studies.

In the evaluation of 3DMPs of PHFs, we obtained a good interobserver correlation in the choice of the surgical treatment, which was statistically significant compared to other traditional methods. Results revealed that ORIF was the most widely used surgical treatment, in line with the literature [24, 30]. Chen et al. [33] confirmed the superiority of 3DPT in terms of the accuracy of fracture characteristics, the intraoperative realization of preoperative planning, and clinical outcomes for complex PHFs. Compared with the conventional method, the 3D printing model for preoperative planning offers shorter operative time, less blood loss, fewer fluoroscopic images, and better functional outcomes.

Limitations of the present study include the unmentioned surgical experience of operators, a small sample of patients, no consideration of intraclass correlation coefficient index, the restricted evaluation of complex PHFs (AO/OTA 11 B and 11 C), and no 3D-CT digital imaging considered.

The CT scan is considered the “gold standard” for PHFs classification systems and decision-making treatment. This study considers both CT imaging and 3DPMs valid and useful for classification systems and treatment choices, even if 3DPMs could help in the surgical managing of complex fracture better than CT scan. 3DPT limitations (time, structure, software, and technical staff dedicated) would be overcome by the increased reproducibility and accuracy in diagnostics of complex fractures like PHFs.

Studies like the study by Brown et al. [23] hypothesized that the 3D modeling will be an effective tool for trauma surgery in the future, which will assist in understanding the complex injury patterns [29, 34–36].

## 5. Conclusion

In the present day, the recent development of 3DPT have a great potential in orthopedics [23]. The application of 3DPMs is superior to X-rays and CT scan regarding the accuracy of the diagnostic evaluation of PHFs and the surgical treatment proposed. Classification systems such as Neer and Hertel have poor reliability between operators in every diagnostic method. It is advisable to use 3DPMs for preoperative fracture planning and modeling patient-specific hardware.

## Figures and Tables

**Table 1:** ICC values interpretation according to Ko and Li guidelines.

ICC values	Reliability grade
<0.5	Poor
0.5–0.75	Moderate
0.75–0.9	Good
>0.9	Excellent

**Table 2:** Neer and Hertel classifications' interobserver reliability.

	Neer classification			Hertel classification		
	X-rays	CT	3DMPs	X-rays	CT	3DMPs
ICC (95% CI)	0.740 (R: 0.342–0.942)	0.792 (R: 0.475–0.946)	0.770 (R: 0.418–0.940)	0.852 (R: 0.625–0.961)	0.806 (R: 0.509–0.949)	0.922 (R: 0.803–0.980)
*P* value	0.002	0.001	0.001	0.001	0.001	0.001

**Table 3:** Treatment and surgical technique' interobserver reliability.

	Treatment indication			Surgery technique		
	X-rays	CT	3DMPs	X-rays	CT	3DMPs
ICC (95% CI)	0.467 (R: −0.349 to −0.861)	1.000 (R: −1.529 to −0.740)	1.000 (R: −1.529 to −0.740)	−0.004 (R: 0.625 to 0.961)	0.429 (R:.−0.444 to −0.851)	0.755 (R: 0.359 to 0.937)
*P* value	0.091	0.451	0.451	0.454	0.116	0.002

## Data Availability

The Excel sheet data used to support the findings of this study are available from the corresponding author upon request (m-acor@hotmail.it). All data generated or analyzed during this study are included in this published article.

## Abbreviations

PHFs: Proximal humeral fractures
CT: Scans computed tomography scan
3DPT: 3D printing technology
IT: Treatment indication
ST: Surgical technique
NC: Neer classification
HC: Hertel classification
ORIF: Open reduction and internal fixation
3DPMs: 3D-printed models
ICC: Intraclass correlation coefficient
CI: Confidence interval.
